# Relationship Between Cardiovascular Disease Pathology and Fatal Opioid and Other Sedative Overdose: A Post-Mortem Investigation and Pilot Study

**DOI:** 10.3389/fphar.2021.725034

**Published:** 2021-11-05

**Authors:** Abdulmalik Zuhair Arab, Aldo Alberto Conti, Fleur Davey, Faisel Khan, Alexander Mario Baldacchino

**Affiliations:** ^1^ Division of Systems Medicine, School of Medicine, University of Dundee, Dundee, United Kingdom; ^2^ Division of Population and Behavioural Science, School of Medicine, University of St Andrews, St Andrews, United Kingdom; ^3^ NHS Fife, Queen Margaret Hospital, Dunfermline, United Kingdom

**Keywords:** CVD (cardiovascular disease), DD (drug death), PMR (post-mortem report), hs-CRP (high-sensitivity C-reactive protein), TNF–α (tumor necrosis factor alpha), IL-6 (interleukin 6), ROS (reactive oxygen species)

## Abstract

**Introduction:** In 2019, Scotland reported the highest number of drug deaths amongst EU countries. Of the 1,264 drug deaths reported in 2019, 94% were related to polysedative use. Studies have proposed a relationship between opioid use and cardiovascular disease. Furthermore, the concomitant use of sedatives and opioids has been associated with lethal cardiopulmonary events. However, evidence is still limited for the relationship between polysedative use and cardiovascular diseases. Thus, the present study aimed to investigate the association between polysedative use and the underlying cardiovascular pathologies in drug deaths.

**Methods:** This study consisted of a post-mortem investigation of 436 drug deaths. Data extracted from post-mortem reports included socio-demographic characteristics (e.g., gender, age), cardiovascular pathologies (e.g., atherosclerosis, atheroma, and inflammation), in addition to the presence of opioids (e.g. methadone, heroin) and other substances (e.g., alcohol, benzodiazepine) in the blood of the deceased. Stepwise multiple regression models were employed to identify which substances predicted cardiovascular pathologies.

**Results:** The presence of opioids, benzodiazepines, and alcohol in the blood of the deceased predicted overall cardiovascular disease (CVD) severity [R2 = 0.33, F (5, 430) = 39.64, *p* < 0.0001; adjusted R2 = 0.32, f2 = 0.49]. Positive Beta coefficients may indicate an exacerbation of CVD (B = 0.48 95% CI = 0.25, 0.70) due to the presence of opioids in the blood of the deceased. Negative associations may instead indicate a relative protective effect of alcohol (B = −0.2, 95% CI = −0.41, −0.00) and benzodiazepines (B = −0.29, 95% CI = −0.48, −0.09) on CVD.

**Conclusion:** These findings may inform national clinical guidelines on the need to monitor individuals who abuse opioids for presence of cardiovascular disease risk factors pathologies and provide timely interventions to reduce mortality in the population.

## Introduction

Substance use is associated with an alarmingly high morbidity and mortality, creating challenges to health care systems around the world (European Monitoring Centre for Drugs and Drug Addiction (EMCDDA), 2021). Drug-related mortality accounts for a substantial percentage of premature deaths in many European countries among high-risk drug users (European Monitoring Centre for Drugs and Drug Addiction (EMCDDA), 2021). In Europe, over 9,200 drug related deaths (DDs) are reported yearly, of which opioid abuse contributes to 80–90% (European Monitoring Centre for Drugs and Drug Addiction (EMCDDA), 2021). In 2019, Scotland reported the highest number of DDs amongst EU countries (National Records of Scotland, 2019). According to The National Records of Scotland (National Records of Scotland, 2019), 94% of DDs consisted of polysedative users.

Specifically, of the 1,264 DDs reported in 2019, 1,205 were related to opioid use (e.g., heroin, morphine, methadone), and 1,040 to additional street and/or prescribed benzodiazepine use (e.g., diazepam, etizolam).

A retrospective cohort study concluded that the leading causes of death related to opioid use are overdose, cardiovascular disease (CVD), cancer, and infectious diseases (Hser et al., 2019). Other studies have linked adverse cardiovascular effects such as coronary heart disease (CHD) (Khodneva et al., 2016), arrhythmia (Doshi et al., 2019a), cardiac arrest (Morrow et al., 2019), and ischemic events (Doshi et al., 2019b) with prescribed opioids and/or opioid overdose.

However, there are conflicting results regarding the relationship between opioid use and CVD. Some studies failed to find any association between chronic opioid use with the increased risk of CVD (Chen and Ashburn, 2015; Chou et al., 2015). A recent survey conducted in 2019, found no association between the use of opioids and CHD (Ogungbe et al., 2019). Interestingly, a review paper suggested a protective role of opioids against myocardial ischemia and reperfusion injury (Tanaka et al., 2014). Other studies investigating the risk of mortality of long-acting opioids prescribed to patients suffering from chronic noncancer pain (Ray et al., 2016) revealed a 1.64 times greater mortality risk compared to matched patients who were prescribed other medication (anticonvulsant or antidepressant) (Ray et al., 2016).

Evidence is also limited about the possible effects of polysedative use on CVD. In fact, while the use of benzodiazepines alone has been associated to reduced risk of CVD (Balon et al., 2018), the concomitant use of benzodiazepines and opioids has been associated with an increased risk of adverse cardiopulmonary events (e.g., respiratory depression) (Yang et al., 2020; Jones et al., 2012). Furthermore, a recent epidemiological study conducted by Tori et al., 2020, in the US revealed a 10.3-fold increase in the mortality rates for opioid overdose deaths involving benzodiazepines, and a 5.5-fold increase in opioid overdose deaths involving alcohol from 1999 to 2017. Indeed, chronic consumption of alcohol has been also associated with a higher risk of CHD (Jalali et al., 2021).

Therefore, there is an urgent need for better understanding the role of opioids and other sedatives in CVD mortality given that opioids, benzodiazepines and alcohol are consumed by individuals with substance use disorder and also by patients admitted with acute myocardial infarction. Additionally, recent studies have suggested that SARS-CoV-2, which causes COVID-19, affects the endothelial system (Sardu et al., 2020), which is a major regulator of cardiovascular health. Specifically, these studies showed that the virus gains entry to host cells via angiotensin-converting enzyme 2 (ACE2), which could cause myocardial dysfunction, plaque instability, microvascular dysfunction, myocardial infraction (MI), and endothelial dysfunction (Guzik et al., 2020). Given the effect of COVID-19 on the endothelium, and the possible increase in long-term CVD morbidity and mortality related to chronic opioid and other sedative use, it is important that this association is studied further to protect patients against the adverse effects of COVID-19 and several other groups of patients who have compromised cardiovascular health. Given the previously quoted studies showing an increase in long-term CVD morbidity related to opioid use, and a synergic effect of opioids and other sedative use on adverse cardiopulmonary events, it is important to better understand this in patients who have CVD. Thus, the present study examines post-mortem data and aims to investigate the association between opioid and other sedative use and the underlying CVD risk factors and pathology in DDs.

## Method

### Cardiovascular Disease Pathologies Classification

Post-mortem reports (PMRs) of individuals deceased between 2013–2019 within the Fife administrative region (Scotland, United Kingdom) with inclusion criteria of DD were anonymized by AB and FD and made available for the study (*n* = 436).

Details of CVD pathologies identified by post-mortem histological examinations were also extracted from each PMR. A total of twelve CVD pathologies were identified by screening all 436 PMRs. These included atherosclerosis (left, right, aorta), atheroma (left, right, aorta), fibrosis, hypertrophy, inflammation, and stenosis (proximal, middle, distal). Pathologies were defined by a Consultant Pathologist based on the histological examination of tissues gained from deceased subjects. A numerical score ranging from 0 to 3 was attributed to each pathology according to its reported severity (0 = No CVD, 1 = Mild, 2 = Moderate, and 3 = Severe). CVD severity was described in each PMR by the Consultant Pathologist who performed the histological examination. To facilitate statistical analyses, severity sub-scores for atherosclerosis (left, right, aorta), atheroma (left, right, aorta), and stenosis (proximal, middle, distal) were combined into a cumulative score for each pathology. Specifically, cumulative severity scores were provided for atheroma, atherosclerosis, and stenosis. These scores ranged from 0 (no CVD) to 9 (severe CVD). Additionally, a “total” CVD severity score was calculated for each PMR by combining the scores of all pathologies (atherosclerosis, atheroma, stenosis, fibrosis, inflammation, hypertrophy). This score ranged from 0 (no CVD) to 36 (severe CVD).

### Research, Ethical and Information Governance Approvals

A request for research access to clinical data relating to the deceased must be treated in the same way as one for data relating to the living. The proposal was considered by NHS Fife Research and Development (R and D) Department, South-East Scotland Regional Ethics Department and NHS Fife Caldicott Guardian on its own merits as per any other project. As our study did not involve NHS clinical time, and the post-mortem data were obtained through a multiagency information sharing memorandum of understanding (2010) to share and disseminate findings of the DDs in Fife in aggregate forms, it was not deemed as research by NHS Fife R and D Department. The secondary analysis of post-mortem results did not need ethical approval. Handling of health-related data of a deceased individual does not need consent, as one relies on other legal bases than consent for processing these data. Whilst the deceased did not have the protection of the Data Protection Act, the advice was that the process of this information should still enshrine a duty of confidentiality, so all the normal data security safeguards were put in place. The study was therefore approved by NHS Fife Caldicott Guardian in 2012.

### Drug Deaths

The definition of a drug death (DD) is complex, with individual studies adopting specific definitions, which vary depending upon the focus of the study. The Scottish Criminal Drugs Enforcement Agency (SCDEA) defines a DD as: ‘‘Where there is prima facie evidence of a fatal overdose of controlled drugs. Such evidence may be recent drug misuse, for example, controlled drugs and/or a hypodermic syringe found in close proximity to the body and/or the person is known to the police as a drug misuser although not necessarily a notified addict.”

The complexity of providing a suitable DD definition is demonstrated by the differences in definitions incorporated by different organisations. For example, the World Health Organisation.

(WHO) defines it as “fatal consequences of the abuse of internationally controlled substances and/or of non-medical use of other substances for psychic effects,” (World Health Organization, 1993). This definition allows the incorporation of deaths indirectly associated with drug abuse, which would be excluded by the SCDEA, such as chronic intoxication, suicide, drug abuse-related accidents, and drug-abuse related diseases. This definition is similar, but not identical, to the definition employed by the General Register Office for Scotland (GROS). The GROS definition includes instances in which toxicological findings indicate the presence of a controlled substance, but where this substance may not necessarily have been a factor contributing to the individual’s death.

### Inclusion and Exclusion Criteria

The inclusion/exclusion criteria presented below incorporates the International Classification of Diseases ICD-10 (F, X, Y) codes used to identify relevant reports for analysis:

Inclusion criteria included:

1) DD, where the underlying cause of death has been coded to the following sub-categories of “mental and behavioural disorders due to psychoactive substance use”: opioids (F11), cannabinoids (F12), sedatives or hypnotics (F13), cocaine (F14), other stimulants, including caffeine (F15), hallucinogens (F16), and multiple drug use and use of other psychoactive substances (F19).
2) deaths coded to the following categories and where a drug listed under the Misuse of Drugs Act (1971) was known to be present in the body at the time of death: accidental poisoning (X40-X44), intentional self-poisoning by drugs, medicaments, and biological substances (X60—X64), assault by drugs, medicaments, and biological substances (X85) and event of undetermined intent, poisoning (Y10-Y14).

Exclusion criteria included:

1) deaths coded to mental and behavioural disorders due to the use of alcohol (F10), tobacco (F17), and volatile substances (F18).
2) deaths coded to drug abuse which were caused by secondary infections and related complications (e.g., septicaemia).
3) deaths from AIDS where the risk factor was believed to be the sharing of needles.
4) deaths where a drug listed under the Misuse of Drugs Act was present because it was part of a compound analgesic or cold remedy, e.g., co-proxamol: paracetamol, dextropropoxyphene or co-dydramol: paracetamol, dihydrocodeine or co-codamol: paracetamol, codeine sulphate as all three of these compound analgesics have, particularly co-proxamol, been used in suicidal overdoses.

### Data Extraction

Data pertaining to relevant socio-demographic characteristics were extracted from each PMR. These included age at the time of death, biological sex, and body mass index (BMI). The presence (or not) of psychoactive substances and/or medicinal drugs, as described in each PMR, was determined by inspecting results of abdominal blood, femoral blood, or ilio-femoral analyses detailed in the toxicology report of each PMR. Substances were grouped into drug classes (opioids, stimulants, anticonvulsants, Tricyclic Antidepressants (TCA), and Selective Serotonin Reuptake Inhibitors (SSRI), benzodiazepines, anticonvulsants, alcohol, and cannabinoids) according to their chemical compounds.

### Statistical Analysis

Frequencies procedures were conducted to determine the percentages (%) of males, females, and of drug classes identified in the sample comprising of 436 deceased individuals. Descriptive statistics were computed to determine the mean and standard deviation (SD) for continuous demographic variables such as age and BMI, and for severity scores of each CVD pathology.

Categorical variables were utilized to determine the presence (or not) of each drug class in post-mortem cases. Specifically, a numerical value of 0 was attributed if concentrations of a particular drug class were not detected by post-mortem blood analyses (e.g., no substances containing opioid compounds such as morphine, codeine, norbuprenorphine, methadone were identified by blood analyses). A numerical value of 1 was instead attributed if concentrations of a particular drug class were detected by post-mortem blood analyses. Drug classifications and categorical values were employed to minimize issues of heterogeneity related to different units of measurements (e.g., mg/dl, g/l), types of substances, and respective metabolites listed in each PMR. A stepwise multiple regression model with a “backward” procedure (Field, 2009) was computed for each CVD pathology and for the total CVD severity score. Thus, seven regression models were computed. This identified which drug classes and demographic variables best predicted CVD severity. Specifically, all drug classes (opioids, stimulants, alcohol, anticonvulsants, cannabis, TCAs, SSRIs, benzodiazepines) and all demographic characteristics (age at the time of death, BMI, biological sex) were included simultaneously in the first step of each regression model as independent variables (IVs).

Females were coded as 0 and males coded as 1. The presence of a drug class was coded as 1 while the absence was coded as 0. Continuous dependent variables (DVs) consisted in the severity scores computed for each pathology (stenosis, atherosclerosis, atheroma, inflammation, hypertrophy, fibrosis) and in the total CVD severity score. The IVs that contributed less to the regression equation (*p* > 0.1) were removed from each model sequentially (Székely et al., 2006; Okamoto et al., 2016). The last step of each regression model included the drug classes and/or the demographic variables that best predicted CVD severity. Statistical significance was set as *p* < 0.05 (Cohen, 1970). Effect sizes (*f* ^2^)for multiple regression models were computed through the software G*Power. The following formula was utilized:

$$f^{2}=\frac{R^{2}}{1-R^{2}}$$ (1)

Where *R* ^2^ = coefficient of multiple determination.

An effect size (*f* ^2^) of 0.02 implies a small effect size, an effect size (*f* ^2^) of 0.15 implies a medium effect size, and an effect size (*f* ^2^) of 0.35 implies a large effect size according to Cohen’s benchmark criteria (Cohen, 1988). Assumptions for stepwise multiple regression models included 1) normally distributed residuals, 2) no multicollinearity, and 3) no highly influential points. The assumption of no highly influential points was assessed by inspecting Cook’s values computed by each regression model (Cook and Weisberg, 1982). A Cook’s value > 1.00 implies a highly influential point. Variance Inflation Factor (VIF) values > 10.00 indicate multicollinearity (Hair et al., 2014). The assumption of normally distributed residuals was assessed by conducting Kolmogorov Smirnoff tests on studentized residuals. A Kolmogorov Smirnoff test result of *p* < 0.05 indicates non-normally distributed residuals. If the assumption of normality was violated, a SQRT transformation was attempted. If the assumption of normality was violated after SQRT transformation, the regression models were still computed. In fact, multiple regression is considered robust against violations of normality when dealing with large sample sizes (>10 observations per variable) (Schmidt and Finan, 2018). As stated by Ernst and Alberts (2017) “the central limit theorem implies that for large samples the sampling distribution of the parameters will be at least approximately normal, even if the distribution of the errors is not. Hence, the regression model is robust with respect to violations of the normality assumption” (Ernst and Albers, 2017). The software SPSS v. 26 (SPSS Inc., United States) was utilized to conduct statistical analyses.

## Results

### Demographics and Cardiovascular Characteristics

Sociodemographic characteristics at the time of death and drug classes identified in the 436 PMRs are depicted in Tables 1, 2. CVD pathologies and their respective severity scores are listed in Table 3.

**TABLE 1:** Demographic characteristics at the time of death and drug classes identified in 436 PMRs.

Variable	N (%)	M	SD	Observed range
Demographics				
Age at the time of death (years)	—	40.0	10.3	18.0–73.0
BMI (kg/m^2^)	—	24.6	6.2	9.9–49.0
Biological sex (Males)	320 (73.4)		—	—
Biological sex (Females)	116 (26.6)		—	—
Drug classes				
Opioids	335 (76.8)		—	—
Stimulants	61 (14.0)		—	—
Alcohol	118 (27.1)		—	—
Cannabinoids	96 (22.0)		—	—
SSRIs	44 (10.1)		—	—
TCAs	74 (17.0)		—	—
Benzodiazepines	150 (34.4)		—	—
Anticonvulsants	96 (22.0)		—	—

Note. SSRI, Serotonin Reuptake Inhibitor; TCA, Tricyclic Antidepressants; N, number of cases; %, percentage; M, Mean; SD, Standard Deviation.

**TABLE 2:** Drug classification based on chemical compounds of substances identified in the 436 PMRs.

Opioids	Stimulants	TCAs	SSRIs	Benzodiazepines	Anticonvulsants
Methadone	Amphetamine	Amitriptyline	Sertraline	Alprazolam	Gabapentin
Buprenorphine	Cocaine	Mirtazapine	Fluoxetine	Diazepam	Pregabalin
Norbuprenorphine	MDMA	—	Citalopram	Etizolam	—
Codeine	—	—	—	—	—
Dihydrocodeine	—	—	—	—	—
Morphine, 6-	—	—	—	—	—
Monoacetylmorphine	—	—	—	—	—
Tramadol, Oxycodone	—	—	—	—	—

Note. TCA, Tricyclic Antidepressants; SSRI, Serotonin Reuptake Inhibitor; MDMA, methylenedioxy-methamphetamine.

**TABLE 3:** Severity scores for cardiovascular pathologies identified in 436 Post-mortem Reports (PMRs).

Cardiovascular pathology	M	SD	Observed range for severity scores	N (%) of PMRs with a severity score ≥1
Atherosclerosis	0.7	1.5	0–9	110 (25.2)
Stenosis	1.5	1.9	0–9	54 (12.3)
Atheroma	0.3	1.1	0–9	243 (55.7)
Inflammation	0.1	0.5	0–3	50 (11.4)
Hypertrophy	0.2	0.6	0–3	69 (15.8)
Fibrosis	0.8	0.9	0–3	226 (51.8)
Total CVD	3.9	3.8	0–22	345 (79.1)

Note. The severity score for atherosclerosis (0 = no CVD to 9 = severe CVD) was obtained by combining scores for left, right, and aorta atherosclerosis. The severity score for atheroma (0 = no CVD to 9 = severe CVD) was obtained by combining scores for left, right, and aorta atheroma. The severity score for stenosis (0 = no CVD to 9 = severe CVD) was obtained by combining scores for proximal, middle, and distal stenosis. The total CVD severity score (0 = no CVD to 36 = severe CVD) was obtained by combining scores for atherosclerosis, stenosis, atheroma, inflammation, hypertrophy, and fibrosis.

M, Mean; SD, Standard deviation; N, number of cases; %, Percentage. PMRs, Post-mortem reports; CVD, cardiovascular disease.

Tables 1, 2 show that most post-mortem cases comprised of middle aged male polysubstance users. BMI data revealed that cases were of normal weight at the time of death according to the Centre for Disease and Control prevention (Centre for Disease Control and Prevention, 2019) cut-off score of <25 kg/m^2^. Mild CVD was identified in most cases (*n* = 345). The most common observed CVD pathologies were atheroma (*n* = 243) and fibrosis (*n* = 226) and the least common were stenosis (*n* = 54) and inflammation (*n* = 50).

### Multiple Regression

Results of the regression model predicting total CVD severity score are depicted in Table 4. Results of regression models predicting the severity of atheroma, fibrosis, atherosclerosis, inflammation, stenosis, and hypertrophy are depicted in Supplementary Tables 1–6.

**TABLE 4:** Stepwise multiple regression model with backward procedure predicting total cardiovascular disease (CVD) severity from age, BMI, sex, opioids, alcohol, cannabis, stimulants, benzodiazepines, TCAs, anticonvulsants, and SSRIs in 436 post-mortem cases.

	B	SE	95% CI	*p*	R	R^2^	Adjusted R^2^
Step 1	—	—	—	—	0.58	0.34	0.32
Constant	−0.72	0.30	−1.31, −0.13	0.017	—	—	—
BMI	0.00	0.00	−0.00, −02	0.35	—	—	—
Age	0.04	0.00	0.03, 0.05	0.00^a^	—	—	—
Biological sex (Male)	0.27	0.11	0.05, 0.49	0.013	—	—	—
Opioids	0.45	0.11	0.21,0.68	0.00^a^	—	—	—
Alcohol	−0.22	0.10	−0.42, −0.01	0.039	—	—	—
Cannabis	−0.07	0.11	−0.30, 0.15	0.52	—	—	—
Stimulants	−0.10	0.13	−0.38, 0.16	0.43	—	—	—
Benzodiazepines	−0.25	0.11	−0.48, 0.02	0.03	—	—	—
TCAs	0.07	0.13	−0.18, 0.32	0.57	—	—	—
Anticonvulsants	−0.10	0.12	−0.35, 0.14	0.40	—	—	—
SSRIs	−0.11	0.14	−0.40, 0.18	0.45	—	—	—
Step 2	—	—	—	—	0.58	0.34	0.32
Constant	−0.72	0.30	−1.31, 0.13	0.017	—	—	—
BMI	0.00	0.00	−0.00, 0.02	0.36	—	—	—
Age	0.04	0.00	0.03, 0.05	0.00^a^	—	—	—
Biological sex (Male)	0.27	0.11	0.05, 0.49	0.014	—	—	—
Opioids	0.45	0.11	0.21, 0.68	0.00^a^	—	—	—
Alcohol	−0.22	0.10	−0.43, 0.01	0.038	—	—	—
Cannabis	−0.06	0.11	−0.28, 0.16	0.58	—	—	—
Stimulants	−0.11	0.13	−0.38, 0.15	0.39	—	—	—
Benzodiazepines	−0.24	0.11	−0.46, −0.01	0.035	—	—	—
Anticonvulsants	−0.09	0.12	−0.34, 0.15	0.44	—	—	—
SSRIs	−0.10	0.14	−0.38, 0.18	0.48	—	—	—
Step 3	—	—	—	—	0.58	0.34	0.32
Constant	−0.76	0.29	−1.33, −0.18	0.01	—	—	—
BMI	0.00	0.00	−0.00, 0.02	0.33	—	—	—
Age	0.04	0.00	0.03, 0.05	0.00^a^	—	—	—
Biological sex (Male)	0.27	0.11	0.05, 0.49	0.014	—	—	—
Opioids	0.45	0.11	0.22, 0.68	0.00^a^	—	—	—
Alcohol	−0.21	0.10	−0.42, −0.00	0.041	—	—	—
Stimulants	−0.11	0.13	−0.38, 0.15	0.39	—	—	—
Benzodiazepines	−0.25	0.11	−0.47, 0.03	0.025	—	—	—
Anticonvulsants	−0.09	0.12	−0.34, 0.15	0.43	—	—	—
SSRIs	−0.09	0.14	−0.38, 0.18	0.49	—	—	—
Step 4	—	—	—	—	0.58	0.34	0.32
Constant	−0.78	0.29	−1.35, −0.20	0.008	—	—	—
BMI	0.00	0.00	−0.00, 0.02	0.32	—	—	—
Age	0.04	0.00	0.03, 0.05	0.00^a^	—	—	—
Biological sex (Male)	0.27	0.11	0.06, 0.49	0.013	—	—	—
Opioids	0.46	0.11	0.23, 0.68	0.00^a^	—	—	—
Alcohol	−0.21	0.10	−0.42, −0.00	0.041	—	—	—
Stimulants	−0.11	0.13	−0.38, 0.15	0.41	—	—	—
Benzodiazepines	−0.25	0.11	−0.47, −0.03	0.022	—	—	—
Anticonvulsants	−0.09	0.12	−0.34, 0.15	0.45	—	—	—
Step 5	—	—	—	—	0.58	0.34	0.32
Constant	−0.77	0.29	−1.35, −0.20	0.008	—	—	—
BMI	0.00	0.00	−0.00, 0.02	0.38	—	—	—
Age	0.04	0.00	0.03, 0.05	0.00^a^	—	—	—
Biological sex (Male)	0.28	0.11	0.06, 0.50	0.010	—	—	—
Opioids	0.46	0.11	0.23, 0.69	0.00^a^	—	—	—
Alcohol	−0.21	0.10	−0.42, −0.00	0.040	—	—	—
Stimulants	−0.11	0.13	−0.38, 0.15	0.39	—	—	—
Benzodiazepines	−0.29	0.09	−0.49, −0.10	0.003	—	—	—
Step 6	—	—	—	—	0.58	0.33	0.32
Constant	−0.82	0.28	−1.39, −0.26	0.004	—	—	—
BMI	0.00	0.00	−0.00, 0.02	0.35	—	—	—
Age	0.04	0.00	0.03, 0.05	0.00^a^	—	—	—
Biological sex (Male)	0.27	0.11	0.06, 0.49	0.012	—	—	—
Opioids	0.47	0.11	0.25, 0.70	0.00^a^	—	—	—
Alcohol	−0.22	0.10	−0.42, −0.01	0.036	—	—	—
Benzodiazepines	−0.29	0.09	−0.49, −0.10	0.003	—	—	—
Step 7	—	—	—	—	0.58	0.33	0.32
Constant	−0.66	0.22	−1.10, −.21	0.004	—	—	—
Age	0.04	0.00	0.04, 0.05	0.00^a^	—	—	—
Biological sex (Male)	0.26	0.10	0.05, 0.48	0.014	—	—	—
Opioids	0.48	0.11	0.25, 0.70	0.00^a^	—	—	—
Alcohol	−0.21	0.10	−0.41, −0.00	0.043	—	—	—
Benzodiazepines	−0.29	0.09	−0.48, −0.09	0.003	—	—	—

Note.

a *p* < 0.0001 level; B, unstandardised beta coefficient; SE, Standard Error; CI, Confidence Interval; R, correlation coefficient; *R* ^2^, coefficient of multiple determination.

SSRI, serotonin selective reuptake inhibitors; BMI, body mass index; TCA, tricyclic antidepressants.

The model including age, biological sex, opioids, alcohol, and benzodiazepines (Step 7) represented the best fit for the regression equation predicting total CVD severity [*R* ^2^ = 0.33, F (5, 430) = 39.64, *p* < 0.0001; adjusted *R* ^2^ = 0.32, *f* ^2^ = 0.49]. Atheroma severity was best predicted by a model including age, opioids, alcohol, and benzodiazepines [*R* ^2^ = 0.22, F (6, 429) = 20.13, *p* < 0.0001; adjusted *R* ^2^ = 0.20, *f* ^2^ = 0.28] (Step 6 in Supplementary Table 1). Similarly, the best regression model predicting fibrosis severity included age, alcohol, and benzodiazepines (Step 9 in Supplementary Figure 2) [*R* ^2^ = 0.14, F (3, 432) = 24.76, *p* < 0.0001; adjusted *R* ^2^ = 0.14, *f* ^2^ = 0.16]. Inflammation was best predicted by a model including age and opioids [*R* ^2^ = 0.02, F (2, 433) = 4.96, *p* < 0.01; adjusted *R* ^2^ = 0.02, *f* ^2^ = 0.02]. However, only opioids predicted inflammation significantly (*p* < 0.05) at the last stage of the backward procedure (Step 10 in Supplementary Table 3).

Hypertrophy severity was predicted significantly by BMI and alcohol (*p* < 0.05) at the last step of the regression model [R2 = 0.02, F (3, 432) = 4.13, *p* < 0.01; adjusted *R* ^2^ = 0.02, *f* ^2^ = 0.02] (Step 9 in Supplementary Table 4). Severity of atherosclerosis was best predicted by age and biological sex [*R* ^2^ = 0.07, F (2, 433) = 16.61, *p* < 0.0001; adjusted *R* ^2^ = 0.06, *f* ^2^ = 0.07] (Step 10 in Supplementary Table 5). Age was instead the only variable to predict significantly (*p* < 0.05) stenosis’ severity [*R* ^2^ = 0.01, F (1, 434) = 6.72, *p* < 0.05; adjusted *R* ^2^ = 0.01, *f* ^2^ = 0.01] (Step 11 in Supplementary Table 6).

By looking at *R* ^2^ statistics and effect sizes described above, it can be noted that total CVD severity score, atheroma, and fibrosis pathologies were strongly influenced by opioids, alcohol, and benzodiazepines. Negative unstandardized Beta coefficients may suggest a relative protective effect of benzodiazepines (B = −0.29, 95% CI = −0.48, −0.09) and alcohol (B = −0.2, 95% CI = −0.41, −0.00) on CVD. On the other hand, positive Beta coefficients may indicate a worsening of CVD due to a higher presence of opioids in the blood of the deceased (B = 0.48, 95% CI = 0.25, 0.70). Opioids were particularly relevant in predicting total CVD score and atheroma severity.

Furthermore, the presence of opioids in post-mortem cases was correlated to cardiovascular inflammation, albeit with a very small effect size (*f* ^2^ = 0.02). Severity of hypertrophy, atherosclerosis, and stenosis were not predicted by any drugs. These pathologies were best predicted by demographic characteristics (e.g., BMI, age, biological sex). However, low *R* ^2^ values and small effect sizes may indicate that other variables not included in the regression models (e.g., diet, genetic susceptibility) may have constituted better predictors.

## Discussion

### Summary of Results

This is the first post-mortem study in the literature, to the best of our knowledge, that investigated the relationship between substance use and CVD pathology in DDs. Our study showed similar descriptive statistics to the 2021 EMCDDA report (European Monitoring Centre for Drugs and Drug Addiction (EMCDDA), 2021) by revealing the presence of opioids in 77% of DDs and therefore, confirming opioid abuse as the fulcrum of Scotland’s drug crisis. Notably, our study revealed an association between the presence of opioids in the system of post-mortem cases and total CVD severity. A relationship was also identified between the presence of opioids and CVD pathologies such as inflammation and atheroma, which are known to lead to atherosclerosis (Hansson, 2009). These findings are consistent with the current literature showing an association between opioid use and poor CVD outcomes (Ziaee et al., 2019). Specifically, a recent literature review conducted by Ziaee et al. (2019) proposed a correlation between chronic opioid use and ischemic stroke. Furthermore, longer duration and higher dosage of opioids were associated with hypertension and with an elevated susceptibility to CHD (Ziaee et al., 2019).

Opioids may have reversed a seemingly cardiovascular protective effect associated to alcohol and benzodiazepines use. Notably, a recent meta-analysis conducted by Yoon et al. (2020) revealed a protective effect of moderate and light alcohol consumption on CVD incidence for individuals aged over 40 years not presenting comorbid conditions. Regarding benzodiazepines, Colussi et al. (2011) revealed low doses of Midazolam to produce a vasodilatation of aortic rings in mice. A retrospective study conducted by Mendelson et al. (2018) revealed chronic benzodiazepines users to present lower blood pressure in comparison to non-benzodiazepines users.

Findings from the current study do not exclude a possible synergic effect of opioids, alcohol, and benzodiazepines on lethal cardiopulmonary events for individuals suffering from CVDs. Indeed, while there is limited and contrasting evidence for the effect of alcohol and benzodiazepines alone on CVD (Balon et al., 2018; Theofilis et al., 2020), the concomitant abuse of alcohol and benzodiazepines may cause cardiovascular and pulmonary toxicity, ultimately leading to cardio-respiratory arrest (Mari et al., 2013).

The proposed synergic effect of opioids and other sedatives on adverse cardiopulmonary events may be in line with a study conducted by Izrailtyan et al. (2018), which showed prescribed opioids and sedatives to be independently associated with the risk of cardiopulmonary arrest in 14,504,809 medical in-patients and 6,771,882 surgical in-patients. The authors also stated that “as compared to patients who received treatment with opioids only, those who received additional sedative medications had a twofold increase in the risk of developing cardiopulmonary arrest” (Izrayltian et al., 2018). Additionally, a recent literature review conducted by Boon et al. (2020) revealed the concomitant use of opioids and benzodiazepines to be associated with an increased risk of suffering lethal respiratory depression in both clinical and non-clinical settings. The above inferences, however, remain highly speculative due to the cross-sectional nature of the current post-mortem study.

The effect of opioids on CVD may have also been influenced by demographic characteristics such as gender and age. In fact, consistently with the literature (North and Sinclair, 2012), older age was predictive of most CVD pathologies (atheroma, fibrosis, atherosclerosis, stenosis).

Notably, an observational cohort study conducted by Gao et al. (2019) revealed that circulatory disease was featured in 11% of all methadone specific DDs occurred in Scotland from 2009 to 2015. Furthermore, circulatory disease was mentioned in 18% of methadone specific DDs occurred at 45 years of age or later. Thus, suggesting a unique adverse effect of methadone on older individuals with underlying CVD pathologies.

The current study did also reveal a significant, albeit small, association between the presence of opioids in post-mortem cases and inflammation severity. Recently, research suggested that chronic inflammation is one the leading causes of cardiac diseases (Fioranelli et al., 2018).

Chronic opioid use has been shown to induce systemic inflammation by exacerbating the up-regulation of pro-inflammatory cytokines such as interleukin 6 (IL-6), C-reactive protein (CRP), and tumour necrosis factor-α (TNF-α) (Lu et al., 2019). Furthermore, the impact of opium addiction on high-sensitivity CRP suggests that opium might cause accelerated multi-system chronic inflammation and coronary atherosclerosis (Reece, 2012). hs-CRP is known to be an important molecular biomarker in activating innate and adaptive immune response to inflammation (Reece, 2012). hs-CRP is mainly produced under the influence of IL-6, and the literature evidence suggests that hs-CRP is correlated with the pathophysiology of atherosclerosis and coronary artery disease (CAD) (Reece, 2012).

Further evidence suggests that opioids can elevate the level and accelerate the formation of reactive oxygen species (ROS) which results in vascular cell damage and endothelial dysfunction (Zahmatkesh et al., 2017).

With the current pandemic of COVID-19, opioids use has also contributed to increasing the risk of COVID-19 infection and the risk of its adverse effects (Wang et al., 2021). Specifically, patients diagnosed with substance use disorders were at about 8 times higher risk of contracting and perishing from COVID-19 compared non-users. This risk was even greater for patients affected by opioid use disorder (Wang et al., 2021). This could be explained by the impact of opioids use on various mechanistic pathways. For instance, considering that opioid use exacerbates the up regulation of IL-6 (Lu et al., 2019), it may worsen the inflammation and consequent cardiovascular outcomes (e.g., myocarditis, plaque rupture) related to the cytokine storm of L-6, IL-7, and IL-22 induced by the viral invasion of SARS-CoV-2.

### Limitations and Strengths

Strengths of the current study include its novelty and its relevance for clinical and public health implications as will be elucidated in the following section. Results from the current study should also be considered in light of several limitations. The first being the lack of a comparable healthy control group comprising of deceased individuals who were not polysedative users. The lack of a comparable group of living polysedative users may also be considered a limitation as such group would have provided context to the limited and opportunistic sample comprising DDs. Methodological limitations include the inability to extract dosage and duration of substance use due to the nature of the information being obtained from routine PMRs.

Furthermore, it was not possible to extract and to statistically control for confounding factors (e.g., diet, physical exercise) which are known to influence cardiovascular health. In fact, by looking at regression models predicting inflammation, hypertrophy, atherosclerosis, and stenosis it could be noted that a high percentage of variance predicting such pathologies remains unexplained (*R* ^2^ values). For example, inflammation may have been also explained by chronic tobacco smoking. In fact, according to a recent meta-analysis conducted by Doggui et al. (2021) there is a robust correlation between chronic tobacco smoking and systemic inflammation (high CRP levels). However, data pertaining to chronic tobacco smoking were not available in post-mortem reports. Additionally, due to the heterogeneity of substances identified in post-mortem cases, it was not possible to investigate the effect of specific substances (e.g., methadone VS buprenorphine) on CVD. Therefore, there is a need for conducting further empirical research investigating the impact of opioids and other sedatives use on CVD in living individuals by taking into-account the above-mentioned limitations.

### Clinical and Public Health Relevance

This post-mortem study revealed a significant positive association between opioids identified in the blood of post-mortem cases and severity of CVD pathologies. Findings from the current study have thepotential to inform national clinical guidelines on the need to monitor individuals who abuse opioids for signs of CVD and provide timely interventions. In fact, the early identification of high risk/at-risk opioid users would contribute to the reduction of early morbidity/mortality in this population. The cardiovascular health of individuals who are prescribed opioids for long term pain relief should also be monitored constantly. Furthermore, considering a possible synergic effect of alcohol, benzodiazepines, andopioids on lethal cardio-pulmonary events, caution should be exercised in prescribing opioids to patients who are heavy alcohol drinkers and/or currently using benzodiazepines.

The burden of worsening CVD outcomes that could be due to chronic opioid use might also have public health consequences for quality of life. One prominent example is the strong association between CVD and depression (Hare et al., 2014). Therefore, healthcare providers should also apply screening tools to assess the psychological burden associated to the development of CVD in high-risk populations such as opioids users. These populations may be also affected by comorbid psychiatric conditions as individuals may utilise substances such as benzodiazepines and opioids concomitantly to self-medicate symptoms of anxiety or mania (Jones et al., 2012; Vogel et al., 2013).

Moreover, given the effect of COVID-19 on the cardiovascular system, and the possible increase in long-term CVD morbidity related to opioids use, it is important that this association is studied further to protect patients affected by COVID-19 and several other groups of patients who have compromised cardiovascular health.

## Conclusion

A significant positive association was identified between opioids use and CVD severity in DDs. These finding could contribute to future evidence-based guidelines indicating more extensive CVD monitoring in those clinical areas working with licit and illicit opioids users. However, additional research into how/why/who/when is affected would improve our understanding of this mechanistic link.

## Data Availability

The raw data supporting the conclusions of this article will be made available by the authors, without undue reservation.
