# Supplementary material for: Relationship Between Cardiovascular Disease Pathology and Fatal Opioid and Other Sedative Overdose: A Post-Mortem Investigation and Pilot Study
Source: Front Pharmacol. 2021 Nov 5;12:725034. doi: 10.3389/fphar.2021.725034 (PMC8602184; doi:10.3389/fphar.2021.725034)
Supplement: Supplementary file 1 [file DataSheet1.docx]

| **Supplementary Table 1.** Stepwise backward multiple regression model (last step) predicting atheroma severity in 436 post-mortem cases from BMI, age, biological sex, opioids, alcohol, stimulants, cannabinoids, SSRIs, TCAs, anticonvulsants, and benzodiazepines | | | | | | |  |
| --- | --- | --- | --- | --- | --- | --- | --- |
|  | B | SE | 95% CI | *p* | R | R^2^ | Adjusted R^2^ |
| **Step 6** |  |  |  |  | 0.46 | 0.22 | 0.20 |
| Constant | -1.71 | 0.40 | -2.50, -0.93 | 0.00* |  |  |  |
| Age | 0.07 | 0.00 | 0.05, 0.08 | 0.00* |  |  |  |
| Biological sex (Male) | 0.34 | 0.18 | -0.01, 0.71 | 0.062 |  |  |  |
| Opioids | 0.43 | 0.02 | 0.02,0.83 | 0.038 |  |  |  |
| Alcohol | -0.44 | 0.19 | -0.81, -0.07 | 0.019 |  |  |  |
| Stimulants | 0.042 | 0.24 | -0.05,0.90 | 0.081 |  |  |  |
| Benzodiazepines | -0.042 | 0.17 | -0.76, -0.07 | 0.016 |  |  |  |
| **Note.***:*p*<0.0001 level; B: unstandardised beta coefficient; SE: Standard Error; CI: Confidence Interval; R: correlation coefficient; R^2^: coefficient of multiple determination. SSRI= serotonin selective reuptake inhibitors; TCA=tricyclic antidepressants; BMI=body mass index | | | | | | | |

| **Supplementary Table 3.** Stepwise backward multiple regression model (last step) predicting inflammation severity in 436 post-mortem cases from BMI, age, biological sex, opioids, alcohol, stimulants, cannabinoids, SSRIs, TCAs, anticonvulsants, and benzodiazepines | | | | | | |  |
| --- | --- | --- | --- | --- | --- | --- | --- |
|  | B | SE | 95% CI | *p* | R | R^2^ | Adjusted R^2^ |
| **Step 10** |  |  |  |  | 0.15 | 0.02 | 0.01 |
| Constant | -0.10 | 0.11 | -0.32,0.11 | 0.35 |  |  |  |
| Age | 0.00 | 0.00 | -0.00,0.01 | 0.09 |  |  |  |
| Opioids | 0.15 | 0.06 | 0.02, 0.27 | 0.021 |  |  |  |
| **Note.** B: unstandardised beta coefficient; SE: Standard Error; CI: Confidence Interval; R: correlation coefficient; R^2^: coefficient of multiple determination. SSRI= serotonin selective reuptake inhibitors; TCA=tricyclic antidepressants; BMI=body mass index | | | | | | | |

| **Supplementary Table 2.** Stepwise backward multiple regression model (last step) predicting fibrosis severity in 436 post-mortem cases from BMI, age, biological sex, opioids, alcohol, stimulants, cannabinoids, SSRIs, TCAs, anticonvulsants, and benzodiazepines | | | | | | |  |
| --- | --- | --- | --- | --- | --- | --- | --- |
|  | B | SE | 95% CI | *p* | R | R^2^ | Adjusted R^2^ |
| **Step 9** |  |  |  |  | 0.38 | 0.14 | 0.14 |
| Constant | -0.15 | 0.17 | -0.49, 0.18 | 0.37 |  |  |  |
| Age | 0.02 | 0.00 | 0.02,0.03 | 0.00* |  |  |  |
| Alcohol | -0.22 | 0.09 | -0.40, -0.03 | 0.019 |  |  |  |
| Benzodiazepines | -0.23 | 0.08 | -0.40,-0.06 | 0.008 |  |  |  |
| **Note.***:*p*<0.0001 level; B: unstandardised beta coefficient; SE: Standard Error; CI: Confidence Interval; R: correlation coefficient; R^2^: coefficient of multiple determination. SSRI= serotonin selective reuptake inhibitors; TCA=tricyclic antidepressants; BMI=body mass index | | | | | | | |

| **Supplementary Table 4.** Stepwise backward multiple regression model (last step) predicting hypertrophy severity in 436 post-mortem cases from BMI, age, biological sex, opioids, alcohol, stimulants, cannabinoids, SSRIs, TCAs, anticonvulsants, and benzodiazepines | | | | | | |  |
| --- | --- | --- | --- | --- | --- | --- | --- |
|  | B | SE | 95% CI | *p* | R | R^2^ | Adjusted R^2^ |
| **Step 9** |  |  |  |  | 0.16 | 0.02 | 0.02 |
| Constant | -0.18 | 0.17 | -0.52, 0.15 | 0.27 |  |  |  |
| BMI | 0.01 | 0.00 | 0.00,0.02 | 0.039 |  |  |  |
| Age | 0.00 | 0.00 | -0.00,0.01 | 0.07 |  |  |  |
| Alcohol | -0.13 | 0.06 | -0.26, -0.00 | 0.048 |  |  |  |
| **Note.** B: unstandardised beta coefficient; SE: Standard Error; CI: Confidence Interval; R: correlation coefficient; R^2^: coefficient of multiple determination. SSRI= serotonin selective reuptake inhibitors; TCA=tricyclic antidepressants; BMI=body mass index | | | | | | | |

| **Supplementary Table 5.** Stepwise backward multiple regression model (last step) predicting atherosclerosis severity in 436 post-mortem cases from BMI, age, biological sex, opioids, alcohol, stimulants, cannabinoids, SSRIs, TCAs, anticonvulsants, and benzodiazepines | | | | | | |  |
| --- | --- | --- | --- | --- | --- | --- | --- |
|  | B | SE | 95% CI | *p* | R | R^2^ | Adjusted R^2^ |
| **Step 10** |  |  |  |  | 0.26 | 0.07 | 0.06 |
| Constant | -1.04 | 0.32 | -1.67,-0.41 | 0.001 |  |  |  |
| Age | 0.03 | 0.00 | 0.02, 0.05 | 0.00* |  |  |  |
| Biological sex (Males) | 0.40 | 0.16 | 0.08, 0.73 | 0.014 |  |  |  |
| **Note.***:*p*<0.0001; B: unstandardised beta coefficient; SE: Standard Error; CI: Confidence Interval; R: correlation coefficient; R^2^: coefficient of multiple determination. SSRI= serotonin selective reuptake inhibitors; TCA=tricyclic antidepressants; BMI=body mass index. | | | | | | | |

| **Supplementary Table 6.** Stepwise backward multiple regression model (last step) predicting stenosis severity in 436 post-mortem cases from BMI, age, biological sex, opioids, alcohol, stimulants, cannabinoids, SSRIs, TCAs, anticonvulsants, and benzodiazepines | | | | | | |  |
| --- | --- | --- | --- | --- | --- | --- | --- |
|  | B | SE | 95% CI | *p* | R | R^2^ | Adjusted R^2^ |
| **Step 11** |  |  |  |  | 0.12 | 0.01 | 0.01 |
| Constant | -0.19 | 0.22 | -0.64,0.24 | 0.37 |  |  |  |
| Age | 0.01 | 0.00 | 0.00,0.02 | 0.01 |  |  |  |
| **Note.** B: unstandardised beta coefficient; SE: Standard Error; CI: Confidence Interval; R: correlation coefficient; R^2^: coefficient of multiple determination. | | | | | | | |
